# Hyperkalaemia-related reduction of RAASi treatment associates with more subsequent inpatient care

**DOI:** 10.1093/ndt/gfae016

**Published:** 2024-01-22

**Authors:** Maria K Svensson, Toyoaki Murohara, Eva Lesén, Matthew Arnold, Thomas Cars, Krister Järbrink, Gengshi Chen, Naru Morita, Sudhir Venkatesan, Eiichiro Kanda

**Affiliations:** Department of Medical Sciences, Renal Medicine, Uppsala University, Uppsala, Sweden; Department of Cardiology, Nagoya University Graduate School of Medicine, Nagoya, Japan; CVRM Evidence, AstraZeneca, Gothenburg, Sweden; Real World Science and Digital, AstraZeneca, Cambridge, UK; Sence Research AB, Uppsala, Sweden; CVRM Evidence, AstraZeneca, Gothenburg, Sweden; Health Economics and Payer Evidence, AstraZeneca, Cambridge, UK; CVRM Medical Affairs, AstraZeneca, Osaka, Japan; Medical and Payer Evidence Statistics, BioPharmaceuticals Medical, AstraZeneca, Cambridge, UK; Department of Medical Science, Kawasaki Medical School, Okayama, Japan

**Keywords:** chronic kidney disease, guidelines, heart failure, hyperkalaemia, renin–angiotensin system

## Abstract

**Background:**

Hyperkalaemia is a barrier to achieving optimal, guideline-directed treatment with renin–angiotensin–aldosterone system inhibitors (RAASis) in patients with chronic kidney disease (CKD) and/or heart failure (HF). This study describes the association between hyperkalaemia-related RAASi treatment reduction and the number of hospitalized days in patients with CKD and/or HF in Sweden and Japan.

**Methods:**

Using data from health registers and hospital medical records, patients with CKD and/or HF currently receiving RAASis who experienced an index hyperkalaemia episode were identified and categorized as having maintained or reduced RAASi treatment post-index; propensity score matching (1:1) was applied to balance the groups in terms of baseline characteristics. Changes in the number of all-cause, CKD- and HF-related hospitalized days per patient-year during 6 months pre- versus post-index and the number of days alive and out of hospital (DAOH) during 6 months post-index were described.

**Results:**

Overall, 20 824 and 7789 patients were included from Sweden and Japan, respectively, 42% and 38% of whom reduced their RAASi treatment after the index hyperkalaemia episode. During the 6 months post-index, all-cause hospitalization increased by 18.2 days [95% confidence interval (CI) 17.0–19.2] per person-year in Sweden and 17.9 days (95% CI 17.4–18.5) per person-year in Japan among patients with reduced RAASi treatment compared with increases of 9.4 days (95% CI 8.6–10.4) and 8.5 days (95% CI 8.0–9.0) per person-year, respectively, among patients with maintained RAASi treatment. The mean DAOH was 121.5 [standard deviation (SD) 75.0] in Sweden and 141.7 (SD 54.5) in Japan among patients with reduced RAASi treatment compared with 154.0 (SD 51.3) and 157.5 (SD 31.6), respectively, among patients with maintained RAASi treatment.

**Conclusion:**

Patients whose RAASi treatment was reduced after a hyperkalaemia episode had more hospitalized days and fewer DAOH compared with patients whose RAASi treatment was maintained.

KEY LEARNING POINTS **What was known:**

- While renin–angiotensin–aldosterone system inhibitors (RAASis) are the cornerstone of treatment for chronic kidney disease (CKD) and heart failure (HF), they also predispose patients to hyperkalaemia, thereby elevating the risk of adverse cardiovascular outcomes and mortality.
- In routine clinical practice, RAASi treatment is commonly down-titrated or discontinued following an episode of hyperkalaemia, thus preventing patients with CKD or HF from receiving the maximum cardiorenal benefits associated with optimal, guideline-directed RAASi treatment.

**This study adds:**

- In this observational cohort study, 42% and 38% of patients with CKD or HF from Sweden (*n* = 20 824) and Japan (*n* = 7789), respectively, had their RAASi treatment reduced following an episode of hyperkalaemia. Propensity score matching was applied to balance these patients with those who maintained RAASi treatment.
- In the following 6 months, patients whose treatment was reduced spent more time in hospital {mean increase 18.2 days [95% confidence interval (CI) 17.0–19.2] per person-year in Sweden, 17.9 days [95% CI 17.4–18.5] per person-year in Japan} than patients whose treatment was maintained [mean increase 9.4 days (95% CI 8.6–10.4) per person-year in Sweden and 8.5 days (95% CI 8.0–9.0) per person-year in Japan].
- Similarly, patients whose treatment was reduced spent fewer days alive and out of hospital {mean 121.5 days [standard deviation (SD) 75.0] in Sweden, 141.7 days [SD 54.5] in Japan} than patients whose treatment was maintained [mean 154.0 days (SD 51.3) in Sweden and 157.5 days (SD 31.6) in Japan].

**Potential impact:**

- Maintaining guideline-directed RAASi treatment is associated with fewer days in hospital and more days spent alive and out of hospital for patients with CKD or HF.
- In line with current international guidelines, novel potassium binder agents may be used to manage episodes of hyperkalaemia and facilitate the continuation of guideline-directed RAASi treatment.

## INTRODUCTION

Renin–angiotensin–aldosterone system inhibitors (RAASis) have been shown to reduce the risk of kidney failure and all-cause mortality in patients with chronic kidney disease (CKD) and of cardiovascular morbidity in patients with CKD or heart failure (HF) [1, 2]. Guidelines recommend treatment with RAASi at the maximum tolerated dose to achieve optimal cardiorenal benefits in these populations [3–8]. However, RAASis also predispose patients to hyperkalaemia by inhibiting production of or increasing resistance to aldosterone [9–11]. The risk of developing hyperkalaemia is twice as high among patients receiving RAASis compared with those not receiving RAASis [12]. If left untreated, hyperkalaemia can increase the risk of adverse outcomes, including cardiac arrhythmias, cardiac arrest and sudden death [13, 14]. This provides a barrier to patients receiving guideline-directed doses aimed at maximizing the cardiorenal benefits of RAASis. As a result, RAASi treatment is often compromised in patients with hyperkalaemia, with down-titration or discontinuation of treatment a common strategy for managing hyperkalaemia in routine clinical practice [15–20]. In Europe, it was found that although 67–93% of patients hospitalized with HF were treated with recommended RAASi agents, only one-third of these received the recommended target dose [21]. A study from the USA found that 38–47% of patients receiving the maximum dose of RAASi had their treatment down-titrated or discontinued following a hyperkalaemia episode [22]. Current international guidelines now recommend novel, oral potassium (K^+^) binder treatments to manage hyperkalaemia and facilitate the continuation of RAASi treatment, with a matching consensus from practising clinicians [3, 23, 24].

Previous research has generally focused on the consequences of suboptimal RAASi dosing [9, 22, 25], and this has been associated with an increased risk of major cardiac and renal events in CKD and HF populations [11, 12, 26]. The inability of some patients to tolerate the maximum target dose due to unrelated adverse events has not received due consideration in studies examining the extent and consequences of suboptimal RAASi dosing. A previous study focusing on patient-level changes in RAASi treatment following a hyperkalaemia episode demonstrated that a hyperkalaemia-related reduction in RAASi was associated with an increased risk of cardiorenal events [20]. The current study was designed to contribute additional perspectives by further investigating patient-level changes in RAASi treatment following a hyperkalaemia episode and aimed to describe the association between hyperkalaemia-related RAASi treatment reduction and healthcare resource utilization in terms of hospitalized days overall and related to CKD or HF, complemented by a patient-oriented measure of days alive and out of hospital (DAOH), in patients with CKD and/or HF in routine clinical practice. Patients were included from routine clinical practice in Sweden and Japan, thus representing two geographically diverse populations with access to different healthcare systems.

## MATERIALS AND METHODS

### Data sources

This observational cohort study used data collected retrospectively from health registers and hospital medical records in Sweden (national registers linked with health records from two large regions) and Japan [Medical Data Vision (MDV)]. The Swedish database includes regional data on laboratory and clinical measurements from electronic health records in two of the largest regions in Sweden (Region Stockholm and Region Skåne) and is linked with three national Swedish registries: the Prescribed Drug Register (with data on filled prescriptions), the National Patient Registry (covering diagnoses and procedures recorded in inpatient and outpatient care settings) and the Cause of Death Registry. All three national registers are held by the Swedish National Board of Health and Welfare, who linked the data sources via unique personal identification numbers. The Japan MDV database captures data from hospitals across Japan, including information on diagnoses, procedures and prescriptions recorded in inpatient and outpatient care settings. Laboratory test results were captured from a subset of hospitals. In Sweden, the study was approved by the Swedish Ethical Review Authority (Dnr 2020-03850). According to the Japanese Ethical Guidelines for Medical and Health Research Involving Human Subjects, ethical approval and informed consent do not apply to the use of de-identified secondary data.

### Study sample

The study sample included patients ≥18 years of age with an index hyperkalaemia episode recorded in an inpatient or specialist outpatient care setting between March 2018 and July 2020 in Sweden or between May 2020 and February 2022 in Japan. Hyperkalaemia was defined in accordance with the International Classification of Diseases 10th Revision (ICD-10) code E87.5 (Japan and Sweden) or as a recorded potassium (K^+^) value >5.0 mmol/l (Sweden only). The index date was the date of the recorded hyperkalaemia diagnosis or K^+^ value >5.0 mmol/l. Hyperkalaemia episodes preceded by <12 months (Sweden) or <6 months (Japan) of available look-back data or those that occurred <6 months before the end of the study period were censored. Patients who died on the day of their hyperkalaemia episode were excluded. Events of hyperkalaemia with laboratory evidence of haemolysis were excluded from the analysis (Sweden only). In cases of multiple eligible hyperkalaemia episodes, the index episode of hyperkalaemia was defined as a randomly selected hyperkalaemia episode that met the above criteria. All included patients were also required to have a recorded diagnosis of CKD [or estimated glomerular filtration rate (eGFR) <60 ml/min/1.73 m^2^ in Sweden) and/or HF up to 6 months (Japan) or anytime (Sweden) before the index date and a filled prescription of at least one RAASi medication up to 90 days (Japan) or up to 120 days (Sweden) before the index date. The RAASi medication classes included angiotensin-converting enzyme inhibitors (ACEis), angiotensin receptor blockers (ARBs), mineralocorticoid receptor antagonists (MRAs) and an angiotensin receptor neprilysin inhibitor (ARNi).

### Maintained versus reduced RAASi

Patients were categorized as having reduced or maintained their pre-hyperkalaemia RAASi treatment based on filled prescriptions, or lack thereof, within the 90 days (Japan) or 120 days (Sweden) before versus after the index hyperkalaemia episode, regardless of whether death occurred within these 90 or 120 days after the index date. Those who did not fill a new prescription for at least one of their pre-index RAASis or had the dose of at least one of their pre-index RAASis reduced by ≥25% were defined as having reduced their RAASi treatment. Those who continued on at least the same dose of their pre-index RAASis were defined as having maintained their RAASi treatment.

Propensity score (PS) matching (1:1) was applied to balance the group who reduced their pre-hyperkalaemia RAASi treatment to those who maintained their RAASi treatment, regarding demographics (age and sex), comorbidities [CKD (overall and by stage), eGFR, dialysis, HF, diabetes, ischaemic heart disease, proteinuria and arrhythmia], baseline comedications [total number of medication classes, use of RAASi (by class and attainment of guideline-directed target dose, and if newly initiated), alpha blockers, beta blockers, beta agonists, K^+^ binders, cardiac glycosides, calcium channel blockers, diuretics (any), loop diuretics, thiazide diuretics, insulin, non-steroidal anti-inflammatory drugs (NSAIDs) and sodium–glucose co-transporter 2 (SGLT2) inhibitors], number of hospitalized days (all-cause and related to CKD, HF or hyperkalaemia) prior to the index date, number of outpatient visits (all-cause and related to CKD, HF or hyperkalaemia) prior to the index date, number of emergency department visits prior to the index date and the index hyperkalaemia event [severity (in the Swedish cohort only), inpatient or outpatient setting, length of hospitalization if applicable]. Logistic regression modelling was used for PS estimation. Callipers of 0.01 (Sweden) and 0.02 (Japan) were used. Covariates with a standardized mean difference (SMD) <0.10 were considered balanced.

### Outcome definitions and statistical analyses

For the primary endpoint analysis, the difference in the number of hospitalized days (per person-years of follow-up) for all-cause, CKD- and HF-related hospitalizations from 6 months before to 6 months after the index hyperkalaemia episode was calculated in the PS-matched cohort who maintained versus reduced their RAASi. We balanced the number of all-cause, CKD- and HF-related hospitalized days prior to the index date between the two groups via PS matching. Hospitalizations were defined as related to CKD and HF based on the presence of recorded CKD or HF diagnosis codes, respectively, in any position.

For the secondary endpoint, the number of DAOH over 6 months from the index hyperkalaemia episode was calculated for each individual patient as follows: total potential patient follow-up time (i.e. 6 months) − (days in hospital + days beyond the date of death until 6 months after the index date). Patients who were lost to follow-up within these 6 months were excluded from these analyses.

### Sensitivity analyses

Patients receiving dialysis are considered a subpopulation of patients with CKD with differing demographic and clinical characteristics compared with those not receiving dialysis. Therefore a separate sensitivity analysis was performed that excluded patients receiving dialysis in the pre-index period. The non-dialysis sensitivity analysis was performed in both the Swedish and Japan cohorts.

Additional sensitivity analyses were performed to determine the impact on the primary endpoint of early deaths in the post-index period and of defining the index date as the discharge date. These sensitivity analyses were performed using the Swedish cohort, as this had the largest sample size and greatest coverage of laboratory data available for PS matching. For the sensitivity analysis excluding early death, patients who died within 120 days after the index hyperkalaemia episode (the period used to categorize patients as having reduced or maintained their RAASi treatment) were excluded to assess the potential impact of early deaths on exposure categorization and the number of hospitalized days during follow-up. In addition, while the type of index hyperkalaemia event (inpatient or outpatient, length of hospitalization if applicable) was included in the PS matching, the potential impact of the length of index hospitalization was further assessed by redefining the index date as the discharge date in patients whose index hyperkalaemia event occurred in the inpatient setting. This latter sensitivity analysis was performed in the population excluding early deaths.

Finally, the extent of residual confounding after PS matching was explored in the Swedish cohort using negative control outcomes as potential indicators of frailty (fracture, inflammatory bowel disease, urinary tract infection and pneumonia). Cox proportional hazards regression analyses were performed to assess the risk of these outcomes in patients who reduced versus maintained treatment (reference). Patients were censored at death or the end of the observation period (31 December 2020). Results were reported with hazard ratios (HRs) and *P*-values.

## RESULTS

### Study sample

In total, 20 824 patients from Sweden and 7789 from Japan were included. The mean age was 76.2 years (Sweden) and 74.6 years (Japan) and 42.5 and 35.4% were female, respectively (Table 1). In Sweden, 86.2% of patients had CKD and 57.0% had HF; in Japan, 49.3% of patients had CKD and 75.0% had HF. The most common RAASi classes in Sweden were ACEis (used by 48.6%), ARBs (43.6%) and MRAs (29.5%). In Japan, ARBs were the most commonly used class (73.4%), followed by MRAs (28.1%) and ACEis (16.8%).

**Table 1:** Patient characteristics at baseline (overall): Sweden and Japan.

	Sweden	Japan
Characteristics	(*n* = 20 824)	(*n* = 7789)
Age at index (years)		
Mean (SD)	76.2 (11.8)	74.6 (12.3)
Median (IQR)	77.5 (70.4–84.5)	76 (69–83)
Female, *n* (%)	8859 (42.5)	2758 (35.4)
Hyperkalaemia severity at index, *n* (%)^a,b^		
Mild	13 286 (64.6)	284 (29.5)
Moderate	4305 (20.9)	349 (36.2)
Severe	2985 (14.5)	330 (34.3)
K^+^ missing	248	6826
Hyperkalaemia setting at index, *n* (%)		
Inpatient	10 230 (40.1)	3109 (39.9)
Outpatient	10 594 (50.9)	4114 (52.8)
Other or missing	0 (0.0)	566 (7.3)
CKD, *n* (%)	17 950 (86.2)	3843 (49.3)
CKD (by stage), *n* (%)^b^		
Stage 3	11 078 (53.2)	236 (26.6)
Stage 4	4744 (22.8)	301 (33.9)
Stage 5	2128 (10.2)	351 (39.5)
eGFR missing	8	6901
Dialysis before index, *n* (%)	1008 (4.8)	1119 (14.4)
HF diagnosis before index, *n* (%)	11 864 (57.0)	5840 (75.0)
Diabetes diagnosis before index, *n* (%)	9496 (45.6)	3287 (42.2)
IHD diagnosis before index, *n* (%)	9066 (43.5)	2835 (36.4)
RAASi, *n* (%)		
ACEi	10 125 (48.6)	1310 (16.8)
ARB	9089 (43.6)	5719 (73.4)
ARNi^c^	528 (2.5)	142 (1.8)
MRA	6151 (29.5)	2186 (28.1)
Newly initiated on RAASi, *n* (%)	1841 (8.8)	1645 (21.1)
Received >1 RAASi, *n* (%)^d^	4860 (23.3)	1517 (19.5)

a Mild, moderate and severe hyperkalaemia equate to potassium values of 5–5.49, 5.5–5.99 and ≥6.0 mmol/l, respectively.

b Percentages calculated after subtracting those with missing data from the denominator.

c Sacubitril cannot be used as a monotherapy in Sweden and Japan (i.e. only as the ARNi form in combination with valsartan).

d Represents classes of RAASi used during the pre-index period. It was not possible to determine whether RAASi therapies were used concurrently or asynchronously during this period.

IHD: ischaemic heart disease.

Overall, 41.9% (*n* = 8716) and 38.2% (*n* = 2976) of patients in Sweden and Japan, respectively, reduced their RAASi treatment after the index hyperkalaemia episode. Following PS matching, the Swedish cohort consisted of 6998 patients in each of the reduced and maintained RAASi groups; the Japanese cohort included 2092 patients in each group. The covariates included in the PS matching are described in Table 2, and all covariates in both Sweden and Japan had an absolute SMD <0.10 after matching. The PS distributions before and after matching are shown in Supplementary Figs. S1 and S2.

**Table 2:** Patient characteristics of groups who reduced versus maintained RAASi, before and after PS matching: Sweden and Japan.

	Sweden: unmatched		Sweden: matched		Japan: unmatched		Japan: matched	
	Reduced	Maintained	Reduced	Maintained	Reduced	Maintained	Reduced	Maintained
Variable	(*n* = 8716)	(*n* = 12 108)	(*n* = 6998)	(*n* = 6998)	(*n* = 2976)	(*n* = 4733)	(*n* = 2092)	(*n* = 2092)
Age at index (years)								
Mean (SD)	76.9 (11.5)	75.7 (11.9)	76.8 (11.6)	76.7 (11.5)	76.7 (11.7)	73.2 (12.5)	75.6 (12.0)	75.7 (11.6)
Median (IQR)	78.3 (71.3–84.5)	77.4 (69.5–84.4)	78.1 (70.5–84.5)	78.4 (70.5–84.5)	78.1 (71.0–85.1)	75.1 (66.9–82.0)	77.4 (69.7–84.1)	77.6 (69.9–83.9)
Female, *n* (%)	3671 (42.1)	5188 (42.8)	2970 (42.4)	3002 (42.9)	1121 (37.7)	1605 (33.9)	757 (36.2)	761 (36.4)
Hyperkalaemia severity at index, *n* (%)^a,b^								
Mild	5065 (58.9)	8221 (68.6)	4272 (61.0)	4466 (63.8)	78 (22.6)	206 (33.5)	51 (21.2)	87 (37.3)
Moderate	2015 (23.4)	2290 (19.1)	1644 (23.5)	1470 (21.0)	99 (28.7)	248 (40.3)	77 (32.0)	87 (37.3)
Severe	1518 (17.7)	1467 (12.2)	1082 (15.5)	1062 (15.2)	168 (48.7)	161 (26.2)	113 (46.9)	59 (25.3)
K^+^ missing	118	130	0	0	2631	4118	1851	1859
CKD, *n* (%)	7665 (87.9)	10 285 (84.9)	6124 (87.5)	6093 (87.1)	1317 (44.3)	2506 (52.9)	957 (45.7)	973 (46.5)
CKD (by stage), *n* (%)^b^								
Stage 3	4241 (48.7)	6837 (56.5)	3605 (51.5)	3565 (50.9)	75 (25.5)	160 (27.1)	59 (28.1)	65 (30.2)
Stage 4	2330 (26.7)	2414 (19.9)	1748 (25.0)	1738 (24.8)	109 (37.1)	191 (32.3)	71 (33.8)	64 (29.8)
Stage 5	1094 (12.6)	1034 (8.5)	771 (11.0)	790 (11.3)	110 (37.4)	240 (40.6)	80 (38.1)	86 (40.0)
eGFR missing	≤5	≤5	0	0	2682	4142	1882	1877
Dialysis before index, *n* (%)	374 (4.3)	634 (5.2)	305 (4.4)	322 (4.6)	359 (12.1)	758 (16.0)	231 (11.0)	218 (10.4)
HF diagnosis before index, *n* (%)	5245 (60.2)	6619 (54.7)	4041 (57.7)	4119 (58.9)	2413 (81.1)	3352 (70.8)	1634 (78.1)	1649 (78.8)
Diabetes diagnosis before index, *n* (%)	3831 (44.0)	5665 (46.8)	3156 (45.1)	3135 (44.8)	1331 (44.7)	1920 (40.6)	936 (44.7)	940 (44.9)
IHD diagnosis before index, *n* (%)	3866 (44.4)	5200 (43.0)	3036 (43.4)	3057 (43.7)	1216 (40.9)	1585 (33.5)	818 (39.1)	849 (40.6)
ACEi, *n* (%)	4317 (49.5)	5808 (48.0)	3401 (48.6)	3440 (49.2)	508 (17.1)	783 (16.5)	369 (17.6)	360 (17.2)
At least 75% of target dose	1580 (18.1)	2015 (16.6)	1234 (17.6)	1218 (17.4)	75 (2.5)	99 (2.1)	49 (2.3)	41 (2.0)
ARB, *n* (%)	3808 (43.7)	5281 (43.6)	3019 (43.1)	2967 (42.4)	2004 (67.3)	3695 (78.1)	1509 (72.1)	1485 (71.0)
At least 75% of target dose	667 (7.7)	800 (6.6)	531 (7.6)	515 (7.4)	566 (19.0)	1137 (24.0)	425 (20.3)	421 (20.1)
ARNi, *n* (%)	287 (3.3)	241 (2.0)	158 (2.3)	169 (2.4)	76 (2.6)	38 (0.8)	31 (1.5)	29 (1.4)
At least 75% of target dose	73 (0.8)	105 (0.9)	53 (0.8)	61 (0.9)	18 (0.6)	13 (0.3)	9 (0.4)	6 (0.3)
MRA, *n* (%)	3270 (37.5)	2881 (23.8)	2232 (31.9)	2313 (33.1)	1355 (45.5)	772 (16.3)	726 (34.7)	701 (33.5)
At least 75% of target dose	683 (7.8)	580 (4.8)	448 (6.4)	466 (6.7)	328 (11.0)	111 (2.4)	128 (6.1)	109 (5.2)
Newly initiated RAASi, *n* (%)	868 (10.0)	973 (8.0)	652 (9.3)	670 (9.6)	714 (24.0)	908 (19.2)	485 (23.2)	437 (20.9)
Hospitalized days in the 6 months before index, mean (SD)								
All-cause	9.7 (14.9)	5.7 (11.2)	7.7 (12.7)	7.6 (13.1)	16.5 (22.0)	10.6 (17.1)	14.0 (19.6)	14.0 (19.6)
Related to HF	4.1 (9.9)	2.2 (6.7)	3.1 (8.4)	3.0 (7.9)	10.8 (19.1)	6.5 (14.5)	9.0 (16.9)	9.2 (17.2)
Related to CKD	2.2 (7.5)	1.3 (5.5)	1.7 (6.4)	1.7 (6.5)	7.0 (15.8)	5.3 (12.7)	6.3 (14.4)	6.3 (14.4)
Length of index hospitalization (days), mean (SD)	6.3 (9.3)	4.1 (6.4)	5.4 (7.6)	5.1 (7.7)	25.8 (32.0)	13.3 (12.0)	16.4 (16.0)	16.1 (13.1)
Number of medication classes before index, mean (SD)	11.3 (4.7)	10.8 (4.7)	11.1 (4.6)	11.2 (4.7)	19.0 (9.5)	16.7 (8.7)	18.3 (9.3)	18.1 (9.3)

The PS matching included covariates presented in the table and the following: diagnosis of proteinuria, arrhythmia, baseline comedications [alpha blockers, beta blockers, beta agonists, K^+^ binders, cardiac glycosides, calcium channel blockers, diuretics (any), loop diuretics, thiazide diuretics, insulin, NSAIDs, SGLT2 inhibitors], last eGFR before/at index, number of hyperkalaemia-related hospitalized days during 6 months before index, number of hospitalized days during the 30 days before index, number of outpatient physician visits during 6 months before index (all-cause, related to HF, CKD, HK), number of emergency department visit 6 months before index. Hyperkalaemia severity was not included in the PS matching of the Japanese cohort due to the extent of missing data.

a Mild, moderate and severe hyperkalaemia equate to K^+^ values of 5–5.49, 5.5–5.99 and ≥6.0 mmol/l, respectively.

b Percentages calculated after subtracting those with missing results from the denominator. Hyperkalaemia severity was not included in the PS-matched Japanese cohort due to the extent of missing data.

The discrepancy between totals and reduced and maintained RAASi cohorts in Japan is caused by missing data on dose precluding classification of some patients as having reduced versus maintained RAASi.

Table 2 presents baseline patient characteristics, RAASi treatment patterns and the number of hospitalized days in the groups who reduced versus maintained their RAASi treatment, before and after PS matching. In Sweden, the mean age was 76.8 and 76.7 years in the PS-matched reduced and maintained groups, respectively; 42.4 and 42.9% were female. In the reduced RAASi group, 87.5% of the patients had CKD compared with 87.1% in the maintained group, and 57.7 and 58.9%, respectively, had HF. Counting patients whose index event occurred in the outpatient setting as 0, the mean length of index hospitalization was balanced between the PS-matched reduced and maintained groups (5.4 and 5.1 days, respectively).

In Japan, the mean age was 75.6 and 75.7 years in the PS-matched reduced and maintained groups respectively; 36.2 and 36.4% were female, 45.7 and 46.5% of the reduced and maintained group recorded CKD at baseline and 78.1 and 78.8% had HF. The mean length of index hospitalization was balanced between the PS-matched reduced and maintained groups (16.4 and 16.1 days, respectively).

### Change in hospitalized days in PS-matched cohorts

**Figure 1:**
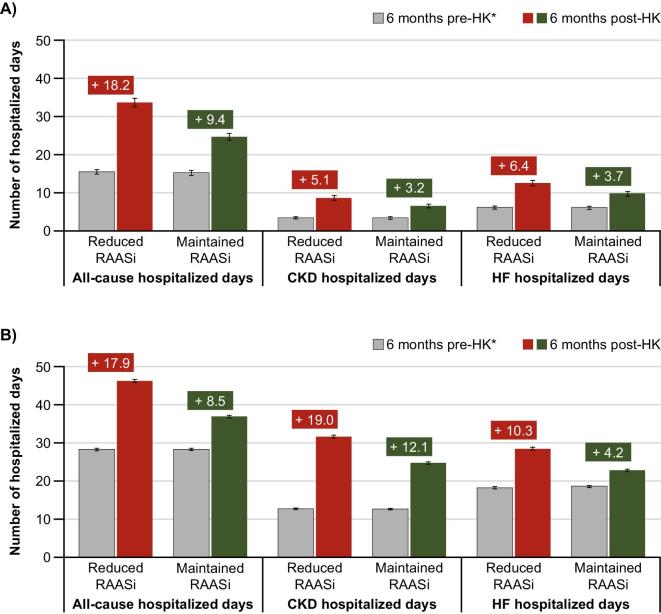
Change in the number of hospitalized days after a hyperkalaemia episode in patients with CKD and/or HF who reduced versus maintained their RAASi treatment following the hyperkalaemia episode in **(A)** Sweden and **(B)** Japan. Error bars show 95% CIs. *1:1 PS matching was applied to balance the cohorts on demographics, comorbidities, baseline comedications (including RAASi) and hospitalized days prior to the index date; the matched cohorts in Sweden included 6998 patients each and in Japan included 2092 patients each. HK: hyperkalaemia.

In the Swedish PS-matched cohorts, the rates of all-cause hospitalized days per person-year during the 6 months prior to index were 15.5 days (95% CI 14.9–16.1) in those who reduced RAASis and 15.2 days (95% CI 14.6–15.8) in those who maintained RAASis. During the 6 months following the index date, the rate per person-year increased by 18.2 days (95% CI 17.0–19.2), to 33.6 days (95% CI 32.5–34.8), in those who reduced RAASis compared with an increase of 9.4 days (95% CI 8.6–10.4), to 24.7 days (95% CI 23.8–25.6), in those who maintained RAASis (Fig. 1A). This corresponds to a 36% higher rate post-index in those who reduced RAASis versus those who maintained RAASis. Similar patterns were observed for CKD- and HF-related hospitalized days; increases of 5.2 days (95% CI 4.6–5.8) and 6.4 days (95% CI 5.7–7.1) were recorded for the reduced RAASi cohort, respectively, compared with 3.1 days (95% CI 2.7–3.6) and 3.7 days (95% CI 3.1–4.3) for the maintained RAASi cohort, respectively.

In the Japanese PS-matched cohorts, the rates of all-cause hospitalized days per person-year in the 6 months prior to the index hyperkalaemia event were 28.3 days (95% CI 27.9–28.6) in those who reduced RAASis and 28.3 days (95% CI 28.0–28.6) in those who maintained RAASis (Fig. 1B). In the 6 months following the index date, the rate per person-year increased by 17.9 days (95% CI 17.4–18.5), to 46.2 days (95% CI 45.8–46.6), in those who reduced RAASis compared with an increase of 8.5 days (95% CI 8.0–9.0), to 36.9 days (95% CI 36.5–37.2) in those who maintained RAASis (Fig. 1B). This corresponds to a 25% higher rate post-index in those who reduced RAASis versus those who maintained RAASis. The corresponding changes in CKD- and HF-related hospitalized days are shown in Fig. 1B.

### Sensitivity analyses

#### Non-dialysis cohort analysis

Overall, 19 568 patients from the Swedish cohort (94.0% of the total cohort) and 6670 from the Japanese cohort (85.6% of the total cohort) were not receiving dialysis in the pre-index period and were included in the non-dialysis cohort sensitivity analysis. Following PS matching, the Swedish cohort consisted of 6667 patients and the Japanese cohort consisted of 1850 patients in each of the reduced and maintained RAASi groups. The PS distributions before and after matching are shown in Supplementary Figs. S3 and S4. Baseline characteristics for patients in the non-dialysis sensitivity analysis are shown in Supplementary Table S1. The change in hospitalized days in the PS-matched cohorts for the reduced versus maintained RAASi treatment groups were consistent with the primary analysis (Supplementary Fig. S5). Rates of all-cause hospitalized days per person-year post-index for the reduced versus maintained RAASi treatment groups were 39% higher in Sweden and 28% higher in Japan (Supplementary Fig. S5).

#### Analysis excluding early deaths

In the Swedish cohort eligible for PS matching (*n* = 20 575), 3959 died within 120 days of the index date. After excluding these patients, 5777 were categorized as having reduced their treatment and 10 839 as having maintained their treatment. The PS distributions before and after matching are shown in Supplementary Fig. S6. In the PS-matched groups (comprising 5103 patients each), the rates of all-cause hospitalized days per person-year post-index were 25.6 days (95% CI 24.3–26.5) and 20.3 days (95% CI 19.5–21.3) in those who reduced and maintained treatment, respectively, corresponding to a 26% higher rate post-index in those who reduced treatment (versus 36% in the main analysis).

#### Discharge index date analysis

The potential impact of the length of index hospitalization was assessed by redefining the index date as the discharge date in patients whose index hyperkalaemia event occurred in the inpatient setting. When the index date was redefined as the date of discharge (where applicable), the rates of all-cause hospitalized days per person-year post-index were 14.3 (95% CI 13.4–14.8) and 11.2 (95% CI 10.5–11.8) in those who reduced and maintained treatment, respectively, corresponding to a 28% higher rate post-index in those who reduced treatment.

### DAOH in PS-matched cohorts

**Figure 2:**
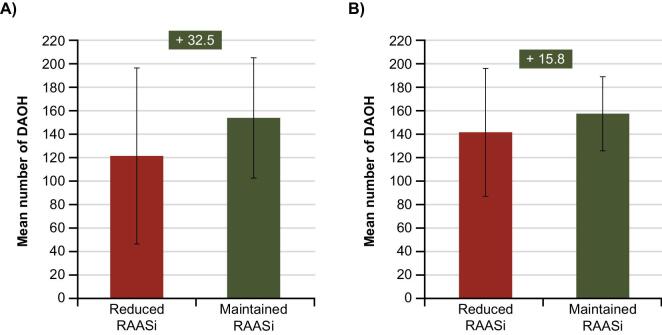
The mean number of DAOH after a hyperkalaemia episode in patients with CKD and/or HF who reduced versus maintained their RAASi treatment following the hyperkalaemia episode in **(A)** Sweden and **(B)** Japan. Error bars show SDs. The number of DAOH over 6 months from the index hyperkalaemia episode was calculated for each individual patient as follows: total potential patient follow-up time (i.e. 6 months) − (days in hospital + days beyond the date of death until 6 months after index). Patients who were lost to follow-up within these 6 months were excluded from these analyses.

Among Swedish patients, those who reduced RAASis recorded a mean of 121.5 DAOH [standard deviation (SD) 75.0] and a median of 168 DAOH [interquartile range (IQR) 27–181]. Those who maintained RAASis recorded a mean of 154.0 DAOH (SD 51.3) and a median of 176 DAOH (IQR 158–183) (Fig. 2A). This trend was also observed in the Japanese PS-matched groups. Among Japanese patients, those who reduced RAASis recorded a mean of 141.7 DAOH (SD 54.5) and a median of 166 DAOH (IQR 134–180). Those who maintained RAASis recorded a mean of 157.5 DAOH (SD 31.6) and a median of 169 DAOH (IQR 150–180) (Fig. 2B).

### Sensitivity analyses on negative control outcomes

Regression analyses exploring the risks of negative control outcomes in those who reduced versus maintained treatment (reference) resulted in HRs of 1.09 (*P* = .261) for fractures, 1.13 (*P* = .770) for inflammatory bowel disease, 0.99 (*P* = .896) for urinary tract infection and 1.09 (*P* = .131) for pneumonia. The Kaplan–Meier curves are shown in Supplementary Fig. S7.

## DISCUSSION

RAASi treatment is a cornerstone of the management of patients with CKD or HF but is often discontinued or down-titrated due to hyperkalaemia, despite guidelines recommending maintained RAASi treatment facilitated by the use of novel oral K^+^ binder treatment [3, 4]. Previous studies have established the association between not achieving the maximum RAASi dose and an elevated risk of adverse clinical outcomes [9, 27].

We hypothesized that following hyperkalaemia-related RAASi treatment reduction, healthcare resource utilization would be higher and there would be fewer DAOH compared with patients who maintained RAASis in contemporary routine clinical practice in two geographically diverse countries with different healthcare systems, Sweden and Japan. The assessment of DAOH serves to add a valuable patient-oriented perspective beyond the measures of healthcare resource utilization and risk of adverse outcomes to assess the potential impact of RAASi reduction [28]. This study showed that patients who had their RAASi treatment reduced had a greater increase in all-cause, CKD- and HF-related hospitalized days and fewer DAOH relative to those who maintained their RAASi treatment. This was true for both the overall cohort including patients receiving dialysis as well as the non-dialysis cohort.

Findings from both countries consistently demonstrated fewer DAOH for patients who reduced versus maintained their RAASi treatment. Despite the relatively short follow-up period of 6 months, the difference in mean DAOH between those who reduced versus maintained RAASi treatment was notable in both countries (121.5 versus 154.0 days in Sweden, 141.7 versus 157.5 days in Japan). The median DAOH did not differ as much between those who reduced and those who maintained their RAASi treatment, especially in Japan (168 versus 176 days in Sweden, 166 versus 169 days in Japan), suggesting that the mean DAOH values may have been elevated by the total number of days lost post-death in those who died during follow-up. However, it should also be noted that the Japanese MDV does not capture deaths outside of the hospital setting. DAOH data pertaining to Japan are therefore likely to be underestimated.

The findings of increased hospitalized days and fewer DAOH associated with reduced RAASi treatment are in alignment with previous studies examining the economic impact of hyperkalaemia in CKD and HF populations, where suboptimal RAASi treatment was associated with an increase in both outpatient visits and overall medical costs [25, 29].

A limitation of the study methodology, albeit an unavoidable one, is the exposure assessment window for categorizing patients as having reduced or maintained their RAASi treatment following the index hyperkalaemia episode. This window (90 or 120 days before and after index) overlapped with the outcome assessment window (6 months after index), therefore imposing a risk of time-related bias. The rationale for this overlap was that even though changes in the RAASi regimen are likely to be implemented soon after the hyperkalaemia episode, the exposure definition is based on filled prescriptions and must therefore allow time for patients to collect a new prescription (or not) and also account for the impact of any prior stockpiling. Patients who died early had an inherently shorter exposure assessment window. In the main analysis, patients who did not refill their RAASi prescription(s) before they died became categorized as having reduced their treatment. This may have diluted the associations since some may in fact have maintained treatment. By contrast, immortal time bias may have been introduced for those who maintained treatment, which may have inflated the associations. In addition, patients who died may have spent more days in hospital prior to their death. The potential impact of these biases was assessed in sensitivity analyses in which patients who died within the 120-day exposure assessment window were excluded and by redefining the index date as the discharge date (where applicable). Compared with the main analysis, rates of hospitalized days during follow-up were lower in the reduced and maintained treatment groups, as anticipated. Nonetheless, the relative magnitude of the increase in hospitalized days among those who reduced versus maintained treatment (26–28% higher) remained comparable to that calculated in the main analysis (36% higher).

While PS matching was applied to balance the groups in terms of baseline covariates, some residual confounding remains likely, with a risk that those who reduced their RAASi treatment had more severe disease or were more frail at baseline compared with those who maintained RAASi treatment, which may have impacted the number of days spent in hospital. Furthermore, additional factors beyond hyperkalaemia may also have influenced the clinician's decision to reduce or maintain RAASis, such as acute kidney failure at the index event. To limit the impact of such confounding, the PS matching also accounted for indicators of severity and frailty, and the matched groups had similar baseline numbers of all-cause as well as CKD- and HF-related hospitalized days and similar numbers of medication classes in total prior to the index hyperkalaemia episode. Furthermore, the sensitivity analysis that assessed the risk of negative control outcomes indicated limited residual confounding.

A strength of this analysis is the inclusion of patients from two different countries. Patients from Sweden and Japan had differences in their baseline demographics (e.g. sex) and characteristics (e.g. underlying rates of CKD and HF) and have access to different healthcare systems and therefore represent two geographically diverse populations. However, there were also important differences between the two datasets used in terms of the type and availability of data. Most notably, practices for identification of hyperkalaemia and CKD differed between the datasets—identification was predominantly based on laboratory values for K^+^ and eGFR in Sweden and ICD-10 diagnosis codes in Japan, leading to missing K^+^ and eGFR data in the Japanese cohort and thus the potential for selection of more severe hyperkalaemia cases for whom a hyperkalaemia diagnosis was recorded. The definitions of CKD- and HF-related hospitalizations may have varying specificity, as they were based on the presence of a recorded CKD or HF diagnosis code in any position. Furthermore, deaths that occurred outside of the hospital setting were not captured in the Japan dataset. Differences in absolute rates of hospitalized days and DAOH between Sweden and Japan are likely due to these country-specific differences. In addition, study periods differed between countries, although they both covered a period when novel K^+^ binders were available and represent the most recent data available at the time of the analyses. Importantly, despite these inherent differences, the trends in increased hospitalized days and fewer DAOH associated with reduced RAASi treatment relative to maintained treatment were consistent between the two countries and datasets.

We found that more than one-third of patients with CKD and/or HF reduced their RAASi treatment after a hyperkalaemia episode. In both Japan and Sweden, reduced RAASi treatment was associated with a greater increase in hospitalized days than maintained RAASi treatment. The mean DAOH were fewer among patients with reduced RAASi treatment than among those who maintained RAASi treatment.

In conclusion, in contemporary routine clinical practice, hyperkalaemia-related reduction in RAASi treatment was associated with a greater increase in the number of hospitalized days and fewer days spent alive and out of hospital compared with maintained RAASi treatment. A better understanding of how adherence to guidelines can be improved to help maintain RAASi treatment following an episode of hyperkalaemia is needed to achieve optimal cardiorenal benefits in patients with CKD or HF.

## Supplementary Material

gfae016_Supplemental_File

## Data Availability

Data underlying the findings described in this article may be obtained from the corresponding author upon reasonable request, in accordance with AstraZeneca's data sharing policy described at https://astrazenecagrouptrials.pharmacm.com/ST/Submission/Disclosure. However, restrictions apply to these data, which were used under license and/or specific approvals for the current study and are not publicly available.

## References

[1] Xie X, Liu Y, Perkovic V et al. Renin-angiotensin system inhibitors and kidney and cardiovascular outcomes in patients with CKD: a Bayesian network meta-analysis of randomized clinical trials. Am J Kidney Dis 2016;67:728–41. 10.1053/j.ajkd.2015.10.011 26597926

[2] Werner C, Baumhakel M, Teo KK et al. RAS blockade with ARB and ACE inhibitors: current perspective on rationale and patient selection. Clin Res Cardiol 2008;97:418–31. 10.1007/s00392-008-0668-3 18454336

[3] Kidney Disease: Improving Global Outcomes Blood Pressure Work Group. KDIGO 2021 clinical practice guideline for the management of blood pressure in chronic kidney disease. Kidney Int 2021;99:S1–87. 10.1016/j.kint.2020.11.003 33637192

[4] McDonagh TA, Metra M, Adamo M et al. 2021 ESC guidelines for the diagnosis and treatment of acute and chronic heart failure. Eur Heart J 2021;42:3599–726. 10.1093/eurheartj/ehab368 34447992

[5] Heidenreich PA, Bozkurt B, Aguilar D et al. 2022 AHA/ACC/HFSA guideline for the management of heart failure: a report of the American College of Cardiology/American Heart Association Joint Committee on Clinical Practice Guidelines. J Am Coll Cardiol 2022;79:e263–421. 10.1016/j.jacc.2021.12.012 35379503

[6] Japanese Society of Nephrology. Essential points from evidence-based Clinical Practice Guidelines for Chronic Kidney Disease 2018. Clin Exp Nephrol 2019;23:1–15. 10.1007/s10157-018-1648-1 30506489 PMC6344397

[7] Tsutsui H, Ide T, Ito H et al. JCS/JHFS 2021 guideline focused update on diagnosis and treatment of acute and chronic heart failure. Circ J 2021;85:2252–91. 10.1253/circj.CJ-21-0431 34588392

[8] National Institute for Health and Care Excellence. Chronic kidney disease: assessment and management. NICE Guideline 203. London: National Institute for Health and Care Excellence, 2021. https://www.nice.org.uk/guidance/ng203 (16 September 2021, date last accessed).

[9] Linde C, Bakhai A, Furuland H et al. Real-world associations of renin-angiotensin-aldosterone system inhibitor dose, hyperkalemia, and adverse clinical outcomes in a cohort of patients with new-onset chronic kidney disease or heart failure in the United Kingdom. J Am Heart Assoc 2019;8:e012655. 10.1161/JAHA.119.012655 31711387 PMC6915283

[10] Viera AJ, Wouk N. Potassium disorders: hypokalemia and hyperkalemia. Am Fam Physician 2015;92:487–95. 26371733

[11] Kohsaka S, Okami S, Morita N et al. Risk–benefit balance of renin–angiotensin–aldosterone inhibitor cessation in heart failure patients with hyperkalemia. J Clin Med 2022;11:5828. 10.3390/jcm11195828. 36233692 PMC9572691

[12] Albasri A, Hattle M, Koshiaris C et al. Association between antihypertensive treatment and adverse events: systematic review and meta-analysis. BMJ 2021;372:n189. 10.1136/bmj.n189 33568342 PMC7873715

[13] Collins AJ, Pitt B, Reaven N et al. Association of serum potassium with all-cause mortality in patients with and without heart failure, chronic kidney disease, and/or diabetes. Am J Nephrol 2017;46:213–21. 10.1159/000479802 28866674 PMC5637309

[14] Nakhoul GN, Huang H, Arrigain S et al. Serum potassium, end-stage renal disease and mortality in chronic kidney disease. Am J Nephrol 2015;41:456–63. 10.1159/000437151 26228532 PMC4686260

[15] Hundemer GL, Talarico R, Tangri N et al. Ambulatory treatments for RAAS inhibitor-related hyperkalemia and the 1-year risk of recurrence. Clin J Am Soc Nephrol 2021;16:365–73. 10.2215/CJN.12990820 33608262 PMC8011018

[16] Ronksley PE, Tonelli M, Manns BJ et al. Emergency department use among patients with CKD: a population-based analysis. Clin J Am Soc Nephrol 2017;12:304–14. 10.2215/CJN.06280616 28119410 PMC5293336

[17] Kashihara N, Kohsaka S, Kanda E et al. Hyperkalemia in real-world patients under continuous medical care in Japan. Kidney Int Rep 2019;4:1248–60. 10.1016/j.ekir.2019.05.018 31517144 PMC6734103

[18] Jackevicius CA, Wong J, Aroustamian I et al. Rates and predictors of ACE inhibitor discontinuation subsequent to elevated serum creatinine: a retrospective cohort study. BMJ Open 2014;4:e005181. 10.1136/bmjopen-2014-005181 PMC4139635 25232564

[19] Wetmore JB, Yan H, Horne L et al. Risk of hyperkalemia from renin-angiotensin-aldosterone system inhibitors and factors associated with treatment discontinuities in a real-world population. Nephrol Dial Transplant 2021;36:826–39. 10.1093/ndt/gfz263 31846025

[20] Kanda E, Rastogi A, Murohara T et al. Clinical impact of suboptimal RAASi therapy following an episode of hyperkalemia. BMC Nephrol 2023;24:18. 10.1186/s12882-022-03054-5 36658531 PMC9854063

[21] Maggioni AP, Anker SD, Dahlstrom U et al. Are hospitalized or ambulatory patients with heart failure treated in accordance with European Society of Cardiology guidelines? Evidence from 12,440 patients of the ESC Heart Failure Long-Term Registry. Eur J Heart Fail 2013;15:1173–84. 10.1093/eurjhf/hft134 23978433

[22] Epstein M, Reaven NL, Funk SE et al. Evaluation of the treatment gap between clinical guidelines and the utilization of renin-angiotensin-aldosterone system inhibitors. Am J Manag Care 2015;21:S212–20. 26619183

[23] Kidney Disease: Improving Global Outcomes Diabetes Work Group. KDIGO 2020 clinical practice guideline for diabetes management in chronic kidney disease. Kidney Int 2020;98:S1–115. 10.1016/j.kint.2020.06.019 32998798

[24] Burton JO, Coats AJS, Kovesdy CP et al. An international Delphi consensus regarding best practice recommendations for hyperkalaemia across the cardiorenal spectrum. Eur J Heart Fail 2022;24:1467–77. 10.1002/ejhf.2612 35791065 PMC9804940

[25] Polson M, Lord TC, Kangethe A et al. Clinical and economic impact of hyperkalemia in patients with chronic kidney disease and heart failure. J Manag Care Spec Pharm 2017;23:S2–9. 10.18553/jmcp.2017.23.4-a.s2 PMC10408394 28436256

[26] Johnson M, Morrison FJ, McMahon G et al. Outcomes in patients with cardiometabolic disease who develop hyperkalemia while treated with a renin-angiotensin-aldosterone system inhibitor. Am Heart J 2023;258:49–59. 10.1016/j.ahj.2023.01.002 36642227

[27] Humphrey TJL, James G, Wittbrodt ET et al. Adverse clinical outcomes associated with RAAS inhibitor discontinuation: analysis of over 400 000 patients from the UK Clinical Practice Research Datalink (CPRD). Clin Kidney J 2021;14:2203–12. 10.1093/ckj/sfab029 34804520 PMC8598122

[28] Fanaroff AC, Cyr D, Neely ML et al. Days alive and out of hospital: exploring a patient-centered, pragmatic outcome in a clinical trial of patients with acute coronary syndromes. Circ Cardiovasc Qual Outcomes 2018;11:e004755. 10.1161/CIRCOUTCOMES.118.004755 30562068 PMC6347414

[29] Neuenschwander JF, Silverstein AR, Teigland CL et al. The increased clinical and economic burden of hyperkalemia in Medicare patients admitted to long-term care settings. Adv Ther 2023;40:1204–23. 10.1007/s12325-022-02420-x 36652174 PMC9988794

