# Supplementary material for: Hyperkalaemia-related reduction of RAASi treatment associates with more subsequent inpatient care
Source: Nephrol Dial Transplant. 2024 Jan 22;39(8):1258–67. doi: 10.1093/ndt/gfae016 (PMC11334062; doi:10.1093/ndt/gfae016)
Supplement: gfae016_Supplemental_File [file gfae016_Supplemental_File.docx]

# Supplementary materials

## Figure S1: Distribution of propensity score (A) before and (B) after matching in Sweden.


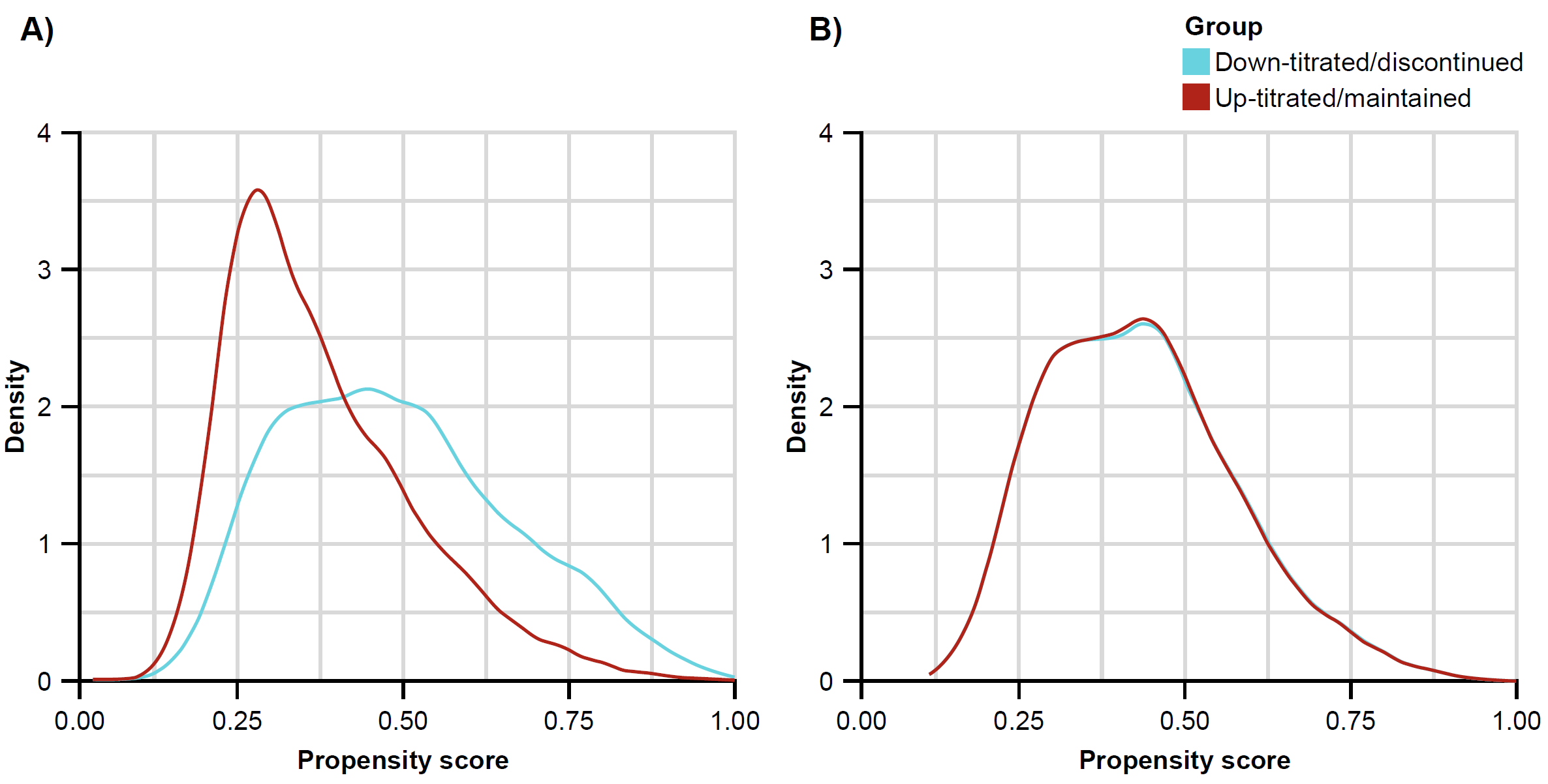


## Figure S2: Distribution of propensity score (A) before and (B) after matching in Japan.


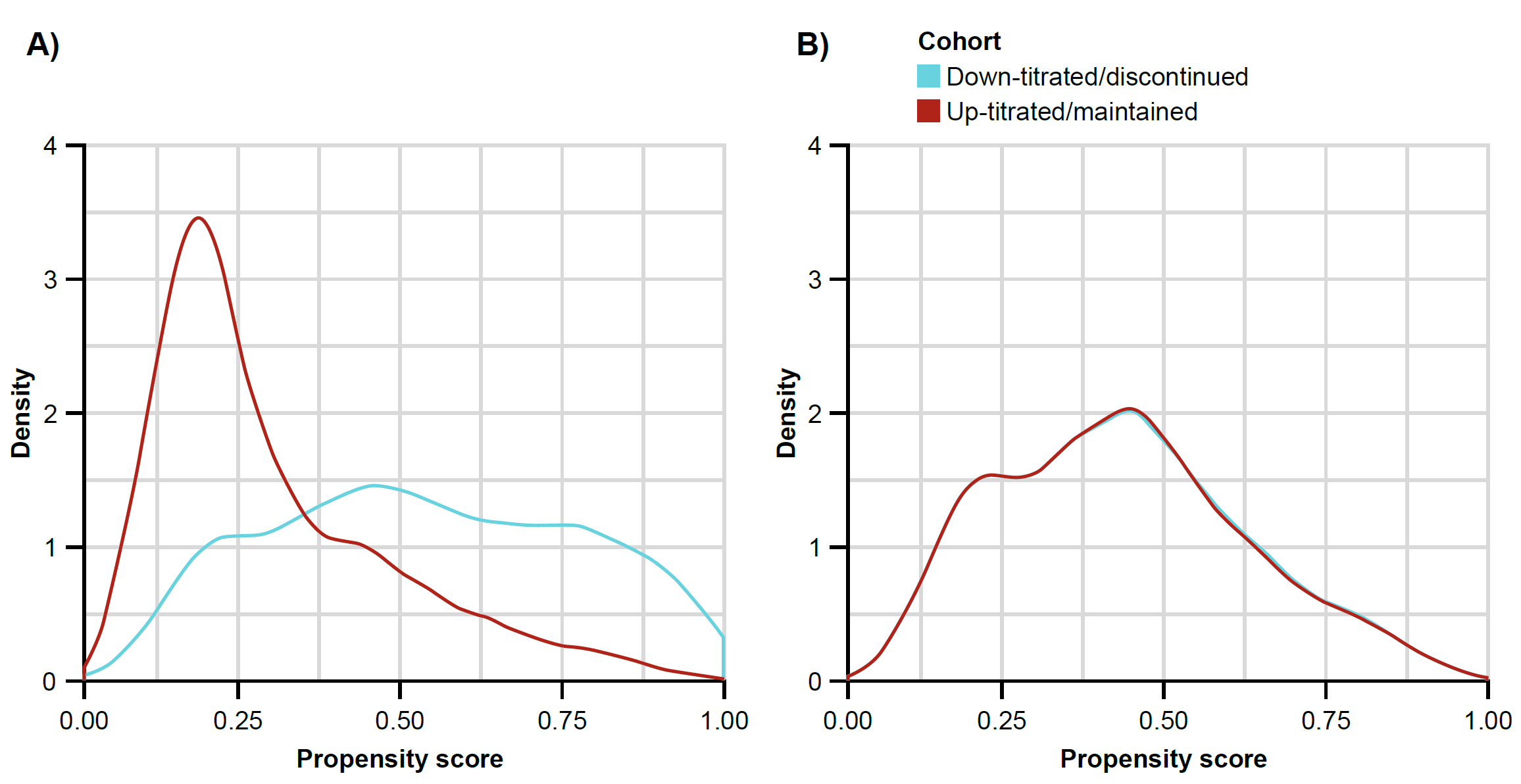


## Figure S3: Sensitivity analysis of non-dialysis patients: Distribution of propensity score (A) before and (B) after matching in Sweden.


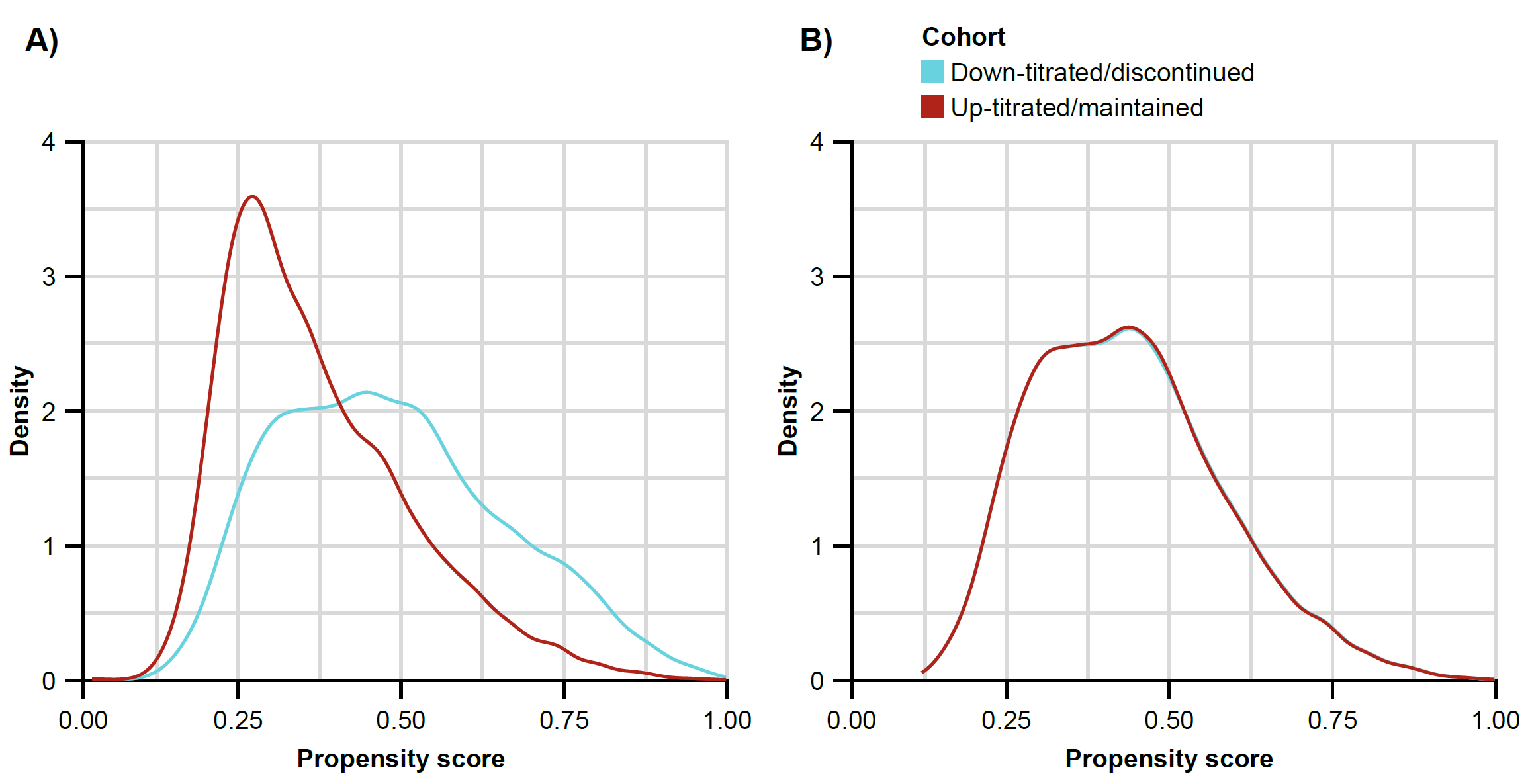


## Figure S4: Sensitivity analysis of non-dialysis patients: Distribution of propensity score (A) before and (B) after matching in Japan.


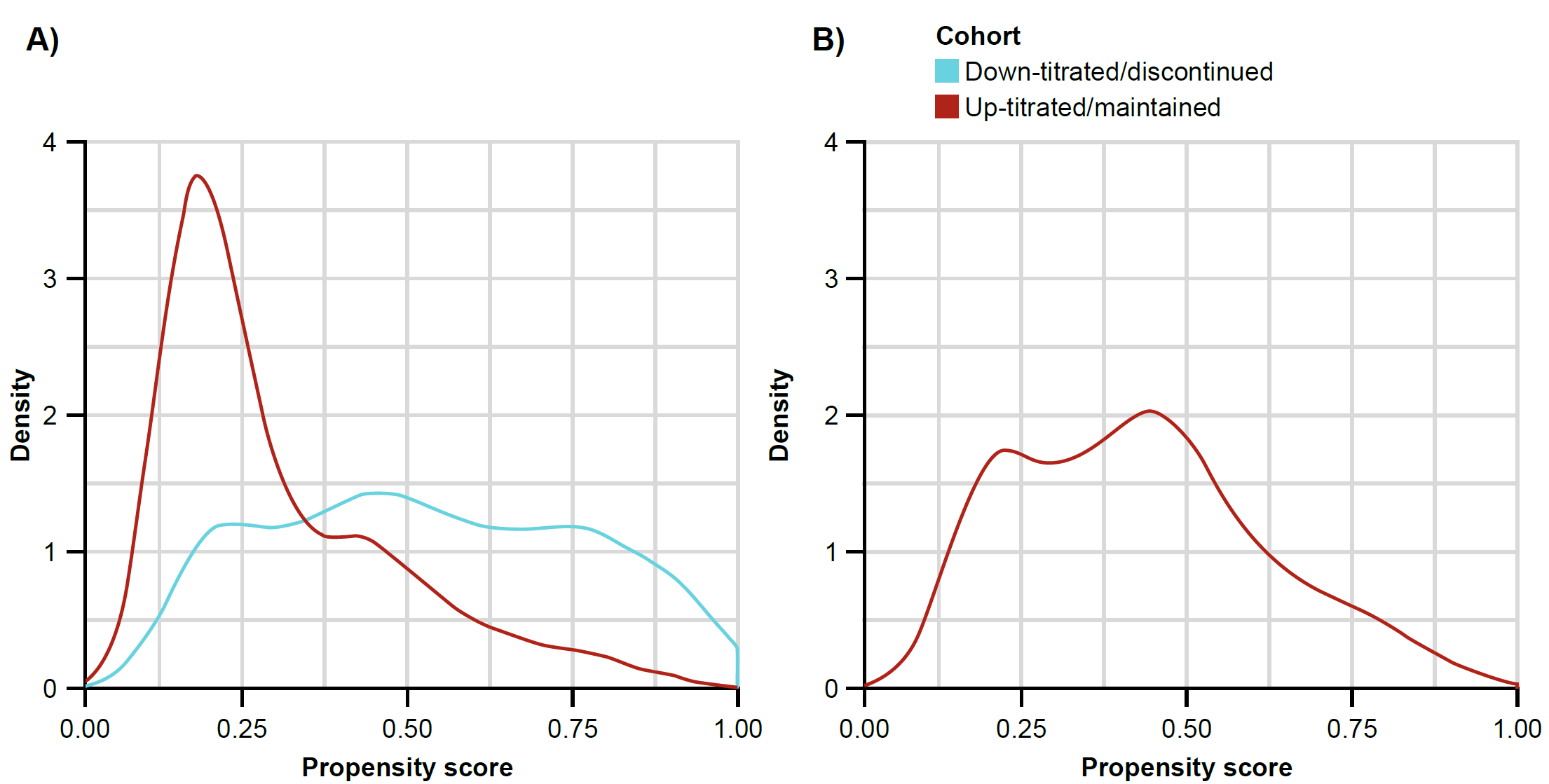


## Figure S5: Change in number of hospitalized days after a hyperkalaemia episode in patients with CKD and/or HF who reduced versus maintained their RAASi treatment following the hyperkalaemia episode in (A) Sweden and (B) Japan (sensitivity analysis excluding patients receiving dialysis pre-index; non-dialysis cohort).

**
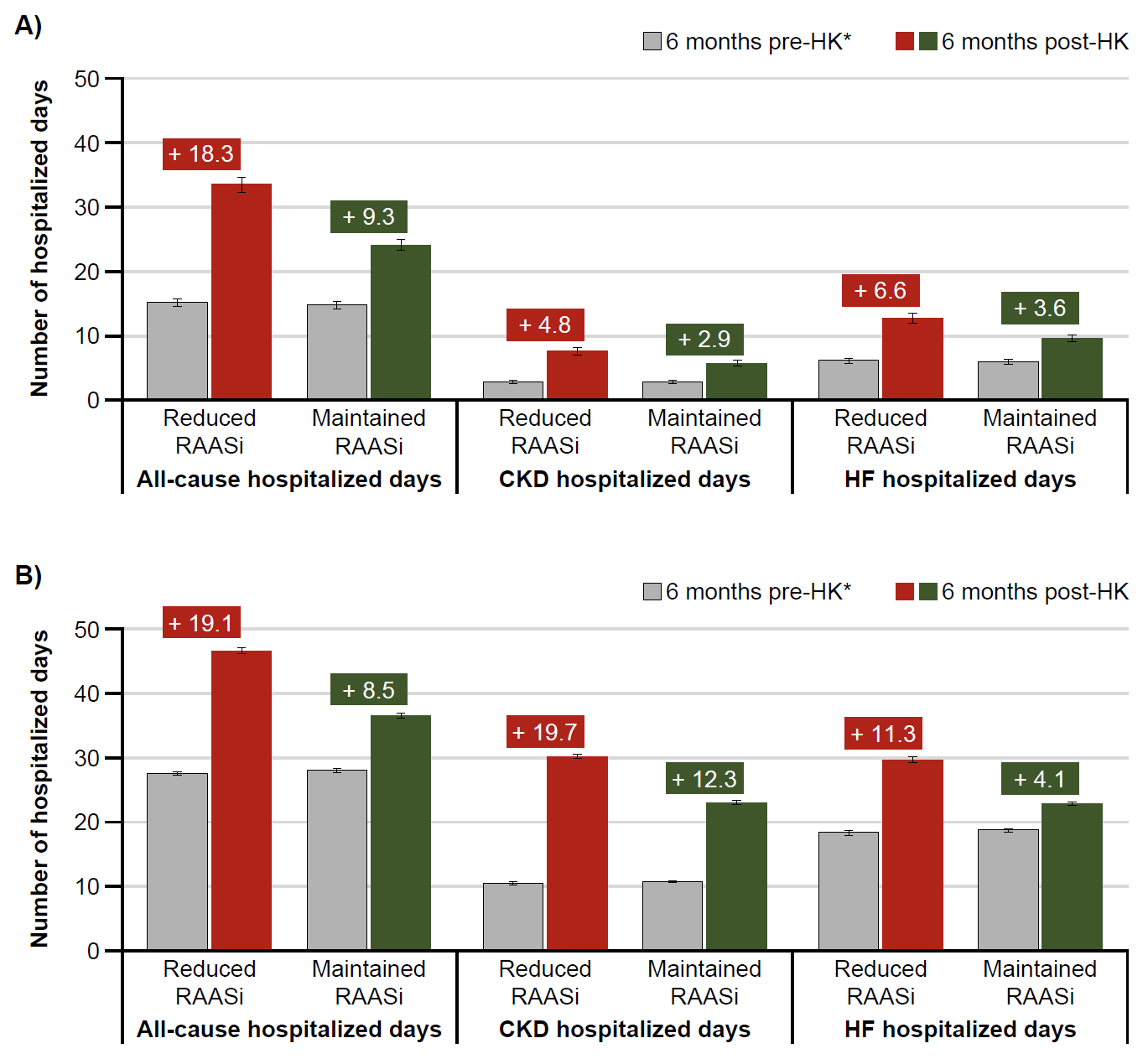
**

Error bars show 95% confidence intervals. *1:1 propensity score matching was applied to balance the cohorts on demographics, comorbidities, baseline comedications (including RAASi), and hospitalized days prior to index.
CKD, chronic kidney disease; HF, heart failure; HK, hyperkalaemia, RAASi, renin–angiotensin–aldosterone system inhibitor.

## Figure S6: Sensitivity analysis excluding early deaths: Distribution of propensity score (A) before and (B) after matching in Sweden.


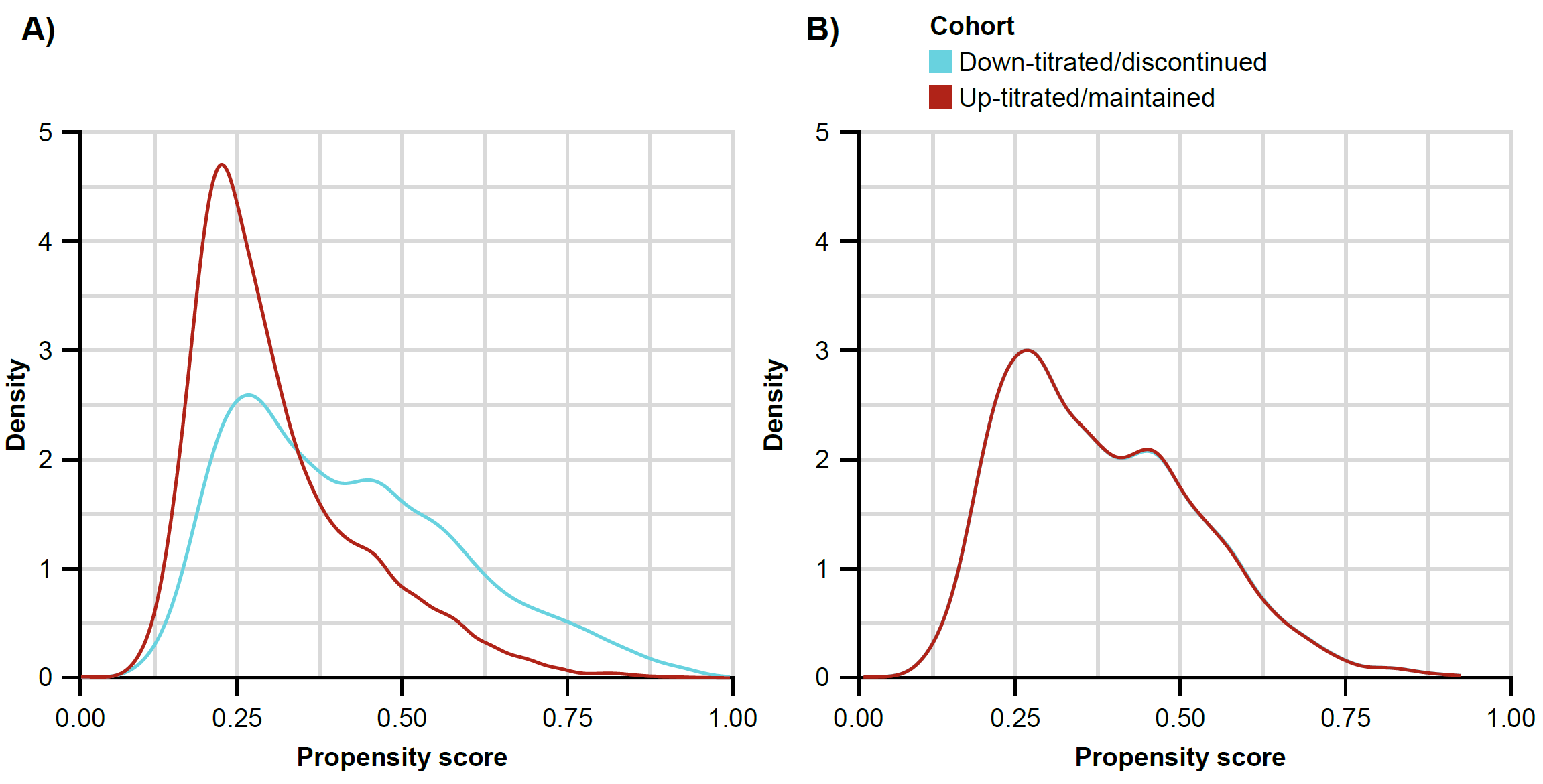


## Figure S7: Kaplan–Meier survival curves for risks of negative control outcomes in those who reduced versus maintained their RAASi treatment (reference) for (A) fractures, (B) inflammatory bowel disease, (C) urinary tract infection and (D) pneumonia in Sweden.

**
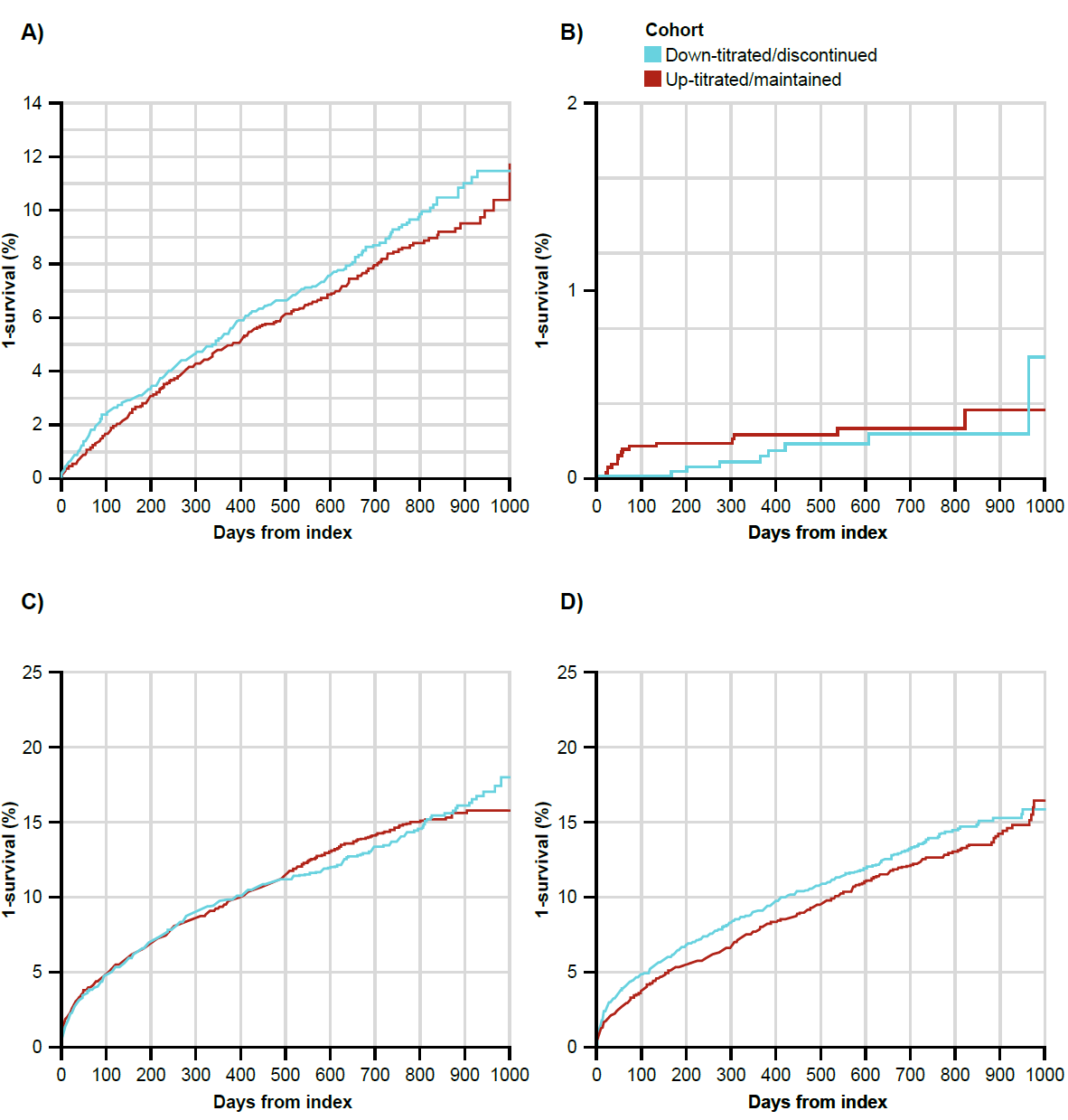
**

Patients were censored at death or the end of the observation period (31 December 2020).
RAASi, renin–angiotensin–aldosterone system inhibitor.

## Table S1: Patient characteristics of groups who reduced versus maintained RAASi, before and after PS-matching (sensitivity analysis excluding patients receiving dialysis pre-index; non-dialysis cohort).

| **Variable** | **Sweden: unmatched** | | **Sweden: matched** | | **Japan: unmatched** | | **Japan: matched** | |
| --- | --- | --- | --- | --- | --- | --- | --- | --- |
|  | **Reduced (*n* = 8342)** | **Maintained (*n* = 11 474)** | **Reduced (*n* = 6667)** | **Maintained (*n* = 6667)** | **Reduced (*n* = 2617)** | **Maintained (*n* = 3975)** | **Reduced (*n* = 1850)** | **Maintained  (*n* = 1850)** |
| Age at index, years |  |  |  |  |  |  |  |  |
| Mean (SD) | 77.5 (11.0) | 76.4 (11.5) | 77.3 (11.1) | 77.3 (11.0) | 77.5 (11.4) | 74.3 (12.2) | 76.6 (11.6) | 76.5 (11.1) |
| Median (IQR) | 78.4 (71.5–85.4) | 77.5 (70.4–84.5) | 78.4 (71.4–85.4) | 78.4 (71.4–85.4) | 79.0 (72.0–86.0) | 76.0 (69.0–83.0) | 78.0 (71.0–85.0) | 78.0 (71.0–84.0) |
| Female, *n* (%) | 3553 (42.6) | 4982 (43.4) | 2868 (43.0) | 2869 (43.0) | 1031 (39.4) | 1349 (33.9) | 693 (37.5) | 661 (35.7) |
| HK severity at index, *n* (%)^a,b^ |  |  |  |  |  |  |  |  |
| Mild | 4853 (58.2) | 7841 (68.3) | 4066 (61.0) | 4272 (64.1) | 64 (21.7) | 175 (32.6) | 39 (18.4) | 71 (35.3) |
| Moderate | 1928 (23.1) | 2134 (18.6) | 1554 (23.3) | 1347 (20.2) | 90 (30.5) | 231 (43.1) | 70 (33.0) | 81 (40.3) |
| Severe | 1444 (17.3) | 1369 (11.9) | 1047 (15.7) | 1048 (15.7) | 141 (47.8) | 130 (24.3) | 103 (48.6) | 49 (24.4) |
| K^+^ missing | 117 (1.4) | 130 (1.1) | 0 | 0 | 2322 | 3439 | 1638 | 1649 |
| CKD, *n* (%) | 7304 (87.6) | 9675 (84.3) | 5794 (86.9) | 5717 (85.7) | 958 (36.6) | 1748 (44.0) | 718 (38.8) | 747 (40.4) |
| CKD (by stage), *n* (%)^b^ |  |  |  |  |  |  |  |  |
| Stage 3 | 4162 (49.9) | 6698 (58.4) | 3535 (53.0) | 3471 (52.1) | 74 (30.2) | 160 (31.7) | 59 (33.1) | 55 (29.9) |
| Stage 4 | 2261 (27.1) | 2310 (20.1) | 1678 (25.2) | 1677 (25.2) | 101 (41.2) | 190 (37.6) | 68 (38.2) | 75 (40.8) |
| Stage 5 | 876 (10.5) | 664 (5.8) | 581 (8.7) | 569 (8.5) | 70 (28.6) | 155 (30.7) | 51 (28.7) | 54 (29.3) |
| eGFR missing | ≤5 | ≤5 | 0 | 0 | 2372 | 3470 | 1672 | 1666 |
| HF diagnosis before index,  *n* (%) | 5092 (61.0) | 6341 (55.3) | 3897 (58.5) | 3976 (59.6) | 2204 (84.2) | 3068 (77.2) | 1510 (81.6) | 1507 (81.5) |
| Diabetes diagnosis before index, *n* (%) | 3662 (43.9) | 5327 (46.4) | 2959 (44.4) | 2975 (44.6) | 1170 (44.7) | 1673 (42.1) | 831 (44.9) | 845 (45.7) |
| IHD diagnosis before index, *n* (%) | 3740 (44.8) | 4972 (43.3) | 2931 (44.0) | 2997 (45.0) | 1082 (41.4) | 1406 (35.4) | 740 (40.0) | 781 (42.2) |
| ACEi, *n* (%) | 4176 (50.1) | 5580 (48.6) | 3270 (49.1) | 3320 (49.8) | 458 (17.5) | 723 (18.2) | 343 (18.5) | 325 (17.6) |
| At least 75% of target dose | 1533 (18.4) | 1958 (17.1) | 1162 (17.4) | 1200 (18.0) | 61 (2.3) | 87 (2.2) | 42 (2.3) | 38 (2.1) |
| ARB, *n* (%) | 3583 (43.0) | 4898 (42.7) | 2819 (42.3) | 2773 (41.6) | 1705 (65.2) | 2992 (75.3) | 1294 (70.0) | 1283 (69.4) |
| At least 75% of target dose | 645 (7.7) | 774 (6.8) | 499 (7.5) | 490 (7.3) | 458 (17.5) | 870 (21.9) | 351 (19.0) | 356 (19.2) |
| ARNi, *n* (%) | 275 (3.3) | 235 (2.1) | 165 (2.5) | 163 (2.4) | 67 (2.6) | 36 (0.9) | 30 (1.6) | 27 (1.5) |
| At least 75% of target dose | 68 (0.8) | 104 (0.9) | 53 (0.8) | 55 (0.8) | 16 (0.6) | 12 (0.3) | 10 (0.5) | 9 (0.5) |
| MRA, *n* (%) | 3214 (38.5) | 2828 (24.7) | 2175 (32.6) | 2246 (33.7) | 1272 (48.6) | 742 (18.7) | 687 (37.1) | 668 (36.1) |
| At least 75% of target dose | 670 (8.0) | 567 (4.9) | 436 (6.5) | 437 (6.6) | 310 (11.9) | 104 (2.6) | 116 (6.3) | 102 (5.5) |
| Newly initiated RAASi, *n* (%) | 838 (10.1) | 925 (8.1) | 640 (9.6) | 653 (9.8) | 651 (24.9) | 813 (20.5) | 443 (24.0) | 400 (21.6) |

The PS matching included covariates presented in the table, as well as the following covariates: diagnosis of proteinuria, arrhythmia, baseline comedications [alpha blockers, beta blockers, beta agonists, potassium binders, cardiac glycosides, calcium channel blockers, diuretics (any), loop diuretics, thiazide diuretics, insulin, NSAIDs, SGLT2 inhibitors], last eGFR before/at index, number of HK-related hospitalized days during 6 months before index, number of hospitalized days during the 30 days before index, number of outpatient physician visits during 6 months before index (all-cause, related to HF, CKD, HK), number of emergency department visit 6 months before index. HK severity was not included in the PS matching of the Japanese cohort due to the extent of missing data.

^a^Mild, moderate and severe HK equate to K^+^ values of 5–5.49, 5.5–5.99, and ≥6.0 mmol/l, respectively.
^b^Percentages calculated after subtracting those with missing results from the denominator. HK severity was not included in the PS-matched Japanese cohort due to the extent of missing data.
The discrepancy between totals and reduced and maintained RAASi cohorts in Japan is caused by missing data on dose precluding classification of some patients as having reduced versus maintained RAASi.

ACEi, angiotensin-converting enzyme inhibitor; ARB, angiotensin-receptor blocker; ARNi, angiotensin receptor neprilysin inhibitor; CKD, chronic kidney disease; eGFR, estimated glomerular filtration rate; HF, heart failure; HK, hyperkalaemia; IHD, ischaemic heart disease; IQR, interquartile range; K^+^, potassium; MRA, mineralocorticoid receptor antagonist; NSAID, non-steroidal anti-inflammatory drug; PS, propensity-score; RAASi, renin–angiotensin–aldosterone system inhibitor; SD, standard deviation; SGLT2, sodium-glucose cotransporter-2.
